# Effect of lower-body ischemia duration in aortic arch surgery under mild-to-moderate hypothermic circulatory arrest

**DOI:** 10.1016/j.xjon.2025.01.015

**Published:** 2025-02-04

**Authors:** Giacomo Murana, Chiara Nocera, Luca Zanella, Luca Di Marco, Silvia Snaidero, Sabrina Castagnini, Carlo Mariani, Davide Pacini

**Affiliations:** a Division of Cardiac Surgery, Cardiac Surgery Department, IRCCS, Azienda Ospedaliero-Universitaria di Bologna, Bologna, Italy; b Department of Medical and Surgical Sciences, DIMEC, Alma Mater Studiorum-University of Bologna, Bologna, Italy

**Keywords:** aorta, aortic arch surgery, circulatory arrest, visceral ischemia

## Abstract

**Objectives:**

Aortic arch surgery is performed at increasingly higher circulatory arrest temperatures. This might affect visceral protection. We analyzed the effect of visceral ischemic time in arch surgery under mild-to-moderate hypothermia.

**Methods:**

We divided the population into 3 groups: group 1 (visceral ischemic time ≤30 minutes), group 2 (31-60 minutes), and group 3 (>60 minutes). The link between visceral ischemic times and in-hospital outcomes, and visceral function biomarker levels were retrospectively analyzed through chi-square test, nonparametric analysis of variance, and cubic spline interpolation.

**Results:**

From 1995 to 2023, 1325 patients underwent aortic arch surgery under circulatory arrest at our center. Mild-to-moderate hypothermia (nasopharyngeal temperature ≥25°) was used in 960 cases. There was no significant difference among the groups for in-hospital death (group 1 = 8.5%, group 2 = 13.2%, group 3 = 11.3%; *P* = .224), renal complications (group 1 = 13.0%, group 2 = 19.7%, group 3 = 22.6%; *P* = .056), and gastrointestinal complications (group 1 = 5%, group 2 = 5.5%, group 3 = 7.1%; *P* = .696). However, respiratory complications (group 1 = 19.4%, group 2 = 28.1%, group 3 = 21.4%; *P* = .027) and transient dialysis (group 1 = 2.8%, group 2 = 7.8%, group 3 = 11.3%; *P* = .011) were linked to longer visceral ischemic times. Groups 2 and 3 presented significantly higher levels of creatinine (*P* < .01), glutamic-oxaloacetic transaminase (*P* < .05), and glutamic pyruvic transaminase (24 and 48 hours postsurgery, *P* < .01). Cubic spline analysis showed that the incidence of renal complications reached a minimum at a low visceral ischemic time and then consistently increased. Respiratory complications showed a maximum incidence at approximately 50 minutes of visceral ischemic time and then subsequently decreased.

**Conclusions:**

Mild-to-moderate hypothermia is a safe strategy for visceral organ protection regardless of visceral ischemic time. However, longer visceral ischemic times are linked to renal complications.

## Graphical Abstract

**Figure:**
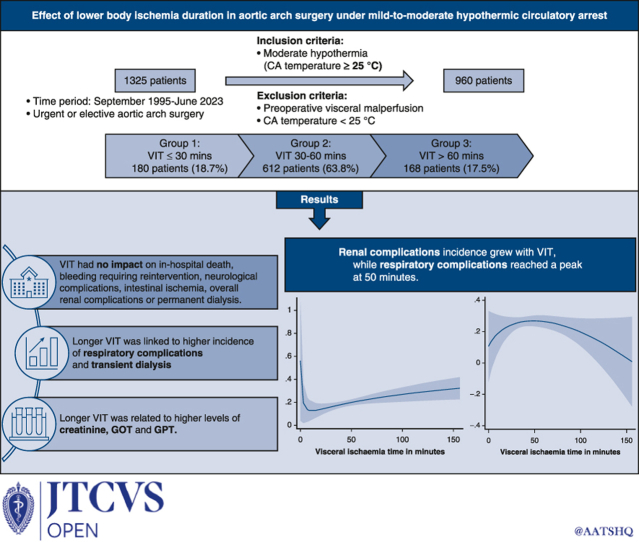

**Figure:**
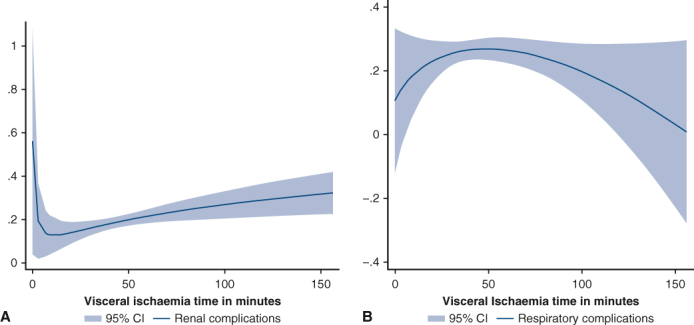
Cubic spline analysis of renal (A) and respiratory (B) complications incidence based on VIT.

**Central Message**

Mild-to-moderate hypothermia is a safe strategy for visceral organ protection regardless of VIT; however, longer VITs are linked to renal and respiratory complications.

**Perspective**

CA is being performed at increasingly higher temperatures because of better cerebral protection techniques. This poses the issue of visceral protection in the context of mild to mild-to-moderate hypothermia. We aimed to study the effect of different VITs on visceral organ function in patients who underwent CA with a nadir temperature greater than 25 °C.

Hypothermic circulatory arrest (CA) provides cerebral and visceral protection in aortic arch surgery. The joint use of hypothermia and antegrade selective cerebral perfusion (ASCP) proved its effectiveness as a brain protection technique.^1^, ^2^, ^3^, ^4^, ^5^ Since its introduction, ASCP allowed a progressive shift toward higher CA temperatures. However, ideal CA temperature remains debated. An international consensus^6^ defined 4 hypothermia categories in aortic arch surgery according to the nasopharyngeal temperature at the beginning of CA:

- Profound: less than 14 °C
- Deep: 14-20 °C
- Moderate: 20-28 °C
- Mild: 28-34 °C

Higher CA arrest temperatures demonstrated good results in terms of mortality and neurological complications, causing a progressive shift toward mild-to-moderate hypothermia.^7^, ^8^, ^9^

Less attention has been given to the effects of mild-to-moderate hypothermia during CA on visceral protection, especially in complex surgeries requiring longer visceral ischemia time (VIT).

The present study aims to evaluate the impact of VIT on visceral organ function among patients who underwent arch surgery under mild-to-moderate hypothermia (≥25 °C).

## Materials and Methods

### Study Population

Between September 1995 and June 2023, 1325 patients underwent elective, urgent, or emergency aortic arch surgery with CA at our center. We chose to examine the cases in which mild-to-moderate hypothermia was used. Moderate hypothermia in this setting is defined as a nasopharyngeal temperature at the beginning of the CA between 20 °C and 28 °C. However, because this is a wide category, we chose to analyze temperatures of 25 °C to 28 °C, which we referred to as “mild-to-moderate hypothermia.”^10^

Our exclusion criteria were the presence of renal or mesenteric malperfusion syndrome^11^^,^^12^ and a CA temperature less than 25 °C.

Hypothermia was combined with ASCP through unilateral or bilateral Kazui. Antegrade cannulation was preferred. When the axillary artery or the brachiocephalic trunk (BCT) was adopted, they were used for cerebral perfusion during CA after clamping of the BCT. In case of femoral cannulation, bilateral Kazui was used through a cannula positioned in the BCT, as well as in the left common carotid artery. Target flow was 10 to 12 mL/kg/min, and near-infrared spectroscopy monitoring was always used.

The patients were divided into 3 groups: group 1 with VIT less 30 minutes, group 2 with VIT 30 to 60 minutes, and group 3 with VIT more than 60 minutes.

The groups were compared in terms of preoperative characteristics, operative strategy, and short-term outcomes. Postoperative visceral function was evaluated by the incidence of renal, gastrointestinal, and respiratory complications, and by the levels of creatinine, glutamic-oxaloacetic transaminase (GOT), glutamic pyruvic transaminase, and bilirubin 24 and 48 hours after surgery, as well as their peak levels (Video 1).

**Figure 1:**
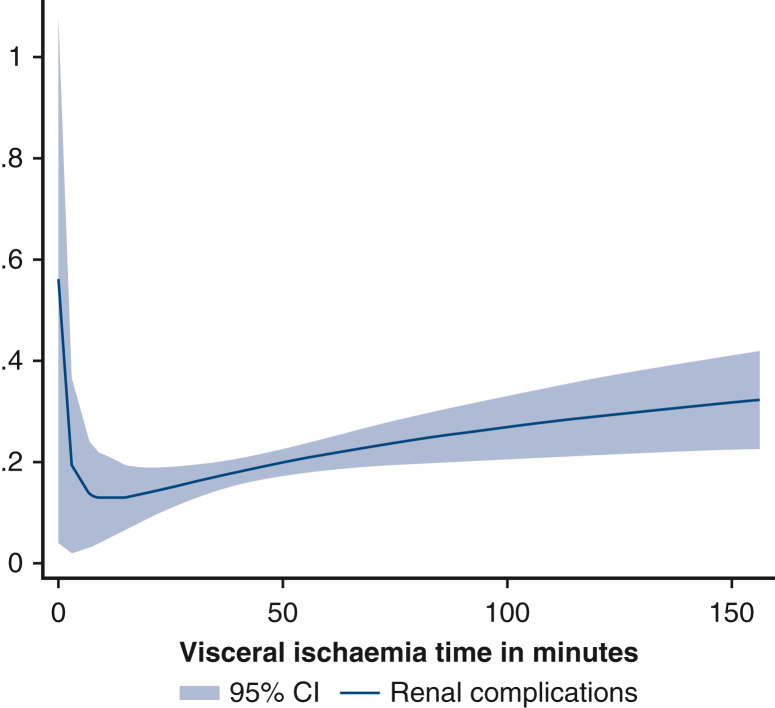
Cubic spline analysis of renal complications incidence according to VIT. *VIT*, Visceral ischemia time.

**Figure 2:**
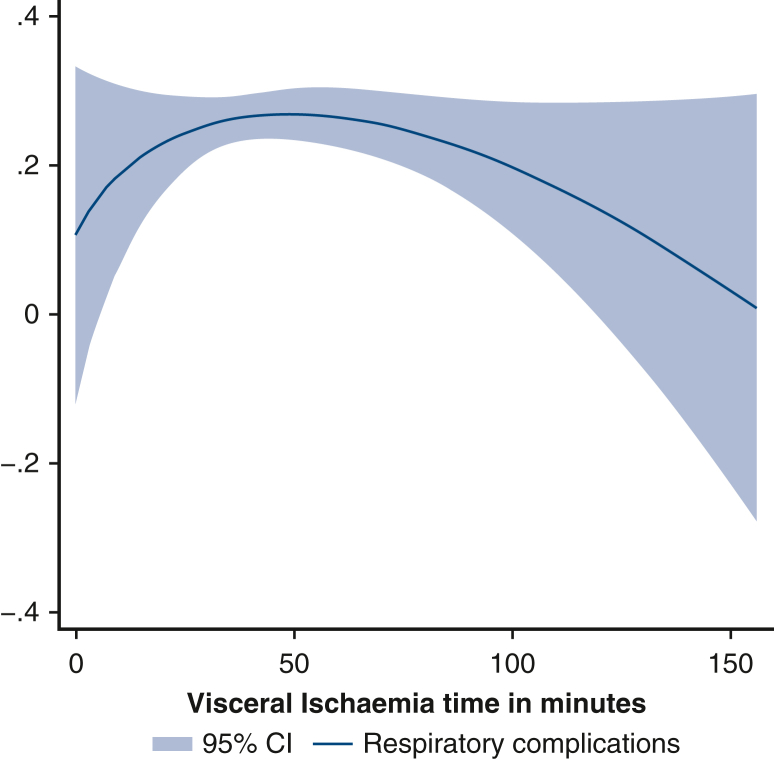
Cubic spline analysis of respiratory complications incidence according to VIT. *VIT*, Visceral ischemia time.

Respiratory complications were defined as intubation time longer than 72 hours or postoperative respiratory failure. Overall renal complications were defined as acute kidney injury (AKI) of any stage according to the RIFLE criteria.

Patients were identified through comprehensive quality registries at IRCCS Azienda Ospedaliero-Universitaria di Bologna. Clinical data were retrieved through medical charts.

These data were approved for the use in human subject by the Institutional Review Board (No. 121/2022/Disp/AUOBo, 11/23), which waived the need for written informed consent.

### Statistical Analysis

Continuous variables were summarized as mean ± SD, and categorical data as absolute numbers and percentages.

Chi-square test and nonparametric analysis of variance (Kruskal–Wallis test) were used to compare the 3 groups in terms of preoperative, intraoperative, and postoperative characteristics, as well as biomarker blood levels.

Logistic regression was used to perform the univariate analysis of risk factors linked to postoperative AKI and respiratory complications. Variables considered clinically relevant for the outcomes were included in the analysis. Afterward, a multivariate logistic regression was performed on the variables that appeared significantly related to the outcome in the univariate analysis.

A cubic spline estimation was carried out to study the probability of renal and respiratory complications depending on VIT. The analysis was graphically represented with the fit fractional polynomial plot method.

The chi-square and analysis of variance tests were performed with SPSS version 26.0 (IBM Corp), and the cubic spline and univariate and multivariate analyses were performed with Stata/SE 17.0 (StataCorp LLC).

## Results

### Study Population

A total of 960 patients represented the study population. Mean VIT was 46.3 ± 20 minutes, and mean nadir nasopharyngeal temperature was 25.4 ± 0.8 °C. Groups 1, 2, and 3 comprised 180 patients (18.7%), 612 patients (63.8%), and 168 patients (17.5%), respectively.

The preoperative characteristics of the 3 groups are depicted in Table 1. Group 2 included more urgent/emergency cases (group 1 = 47, 26.1%; group 2 = 282, 46%; group 3 = 66, 39.3%, *P* < .01). Type A aortic dissection (TAAD) was significantly more frequent in groups with higher VITs (group 1 = 47, 26.1%; group 2 = 232, 37.9%%; group 3 = 54, 32.2%, *P* = .03), and aneurysms were more frequent in group 1 (group 1 = 119, 66.1%; group 2 = 288, 47%; group 3 = 87, 51.7%, *P* < .01). There was no significant difference in preoperative risk factors among the 3 groups.

**Table 1:** Preoperative characteristics of the patients according to the visceral ischemia time

Preoperative characteristics	Overall (n = 960)	VIT ≤30 min (n = 180)	VIT 30-60 min (n = 612)	VIT >60 min (n = 168)	*P* value
Mean age, y	63.4 ± 12	62.6 ± 12.3	64.1 ± 11.4	61.5 ± 13.6	**.021**
Urgent/emergency surgery	395 (41.1%)	47 (26.1%)	282 (46%)	66 (39.3%)	**<.01**
Indication					
Type A aortic dissection	333 (34.7%)	47 (26.1%)	232 (37.9%)	54 (32.2%)	**.003**
Intramural hematoma	49 (5.1%)	5 (2.8%)	36 (5.9%)	8 (4.8%)	.248
Penetrating ulcer	20 (2.1%)	5 (2.8%)	13 (2.1%)	2 (1.2%)	.582
Aneurysm	494 (51.4%)	119 (66.1%)	288 (47%)	87 (51.7%)	**<.01**
Other	64 (6.7%)	4 (2.2%)	43 (7.1%)	17 (10.1%)	.074
Preoperative renal failure	59 (6.1%)	13 (7.2%)	33 (5.4%)	13 (7.7%)	.442
Preoperative dialysis	9 (0.9%)	1 (0.5%)	5 (0.8%)	3 (1.8%)	.436
Diabetes	58 (6%)	14 (7.8%)	34 (5.6%)	10 (6%)	.555
Smoking	372 (38.7%)	68 (37.8%)	234 (38.2%)	70 (41.7%)	.702
Hypertension	692 (72.1%)	122 (67.8%)	452 (73.9%)	118 (70.3%)	.205
Marfan syndrome	30 (3.1%)	2 (1%)	22 (3.6%)	6 (3.6%)	.223
Previous cardiac surgery	279 (29.1%)	51 (28.3%)	182 (29.7%)	46 (27.4%)	.814
Preoperative creatinine levels (mg/dL)	1.12 ± 0.54	1.07 ± 0.7	1.1 ± 0.45	1.22 ± 0.63	**.03**

Bold value indicates are statistically significant. *VIT*, Visceral ischemia time.

### In-Hospital Outcomes

Longer VITs were linked to more complex surgeries, less hemiarch (group 1 = 92, 51.1%; group 2 = 252, 41.2%; group 3 = 32, 19.1%; *P* < .01) and more arch replacement (group 1 = 53, 29.4%; group 2 = 132, 21.6%; group 3 = 58, 34.5%, *P* = .03), frozen elephant trunk (FET) (group 1 = 27, 15%; group 2 = 201, 32.8%; group 3 = 62, 36.9%, *P* < .01), and elephant trunk (ET) (group 1 = 8, 4.5%; group 2 = 27, 4.4%; group 3 = 16, 9.5%, *P* < .01). Group 1 presented more aortic cannulations and less femoral, BCT, and carotid cannulations (Table 2). Mean Kazui flow was 11.65 ± 2.04 mL/kg/min.

**Table 2:** Intraoperative characteristics of the patients according to the visceral ischemia time

Intraoperative characteristics	Overall (n = 960)	VIT ≤30 min (n = 180)	VIT 30-60 min (n = 612)	VIT >60 min (n = 168)	*P* value
CPB times	210.2 ± 64.5 min	186.3 ± 64.7 min	210.6 ± 60 min	234.6 ± 70.8 min	**<.01**
Crossclamp times	135.4 ± 49.2 min	121.6 ± 48.4 min	133.6 ± 46.6 min	156.8 ± 52.9 min	**<.01**
Surgical procedures					
Bentall	315 (32.8%)	71 (49.4%)	201 (32.8%)	43 (25.6%)	**.001**
Hemiarch replacement	376 (39.2%)	92 (51.1%)	252 (41.2%)	32 (19.1%)	**<.01**
Arch replacement	243 (25.3%)	53 (29.4%)	132 (21.6%)	58 (34.5%)	**.03**
FET	290 (30.2%)	27 (15%)	201 (32.8%)	62 (36.9%)	**<.01**
ET	51 (5.3%)	8 (4.5%)	27 (4.4%)	16 (9.5%)	**<.01**
Cannulation site					
Aortic	145 (15.1%)	49 (27.2%)	77 (12.6%)	19 (11.3%)	**<.01**
Axillary	254 (26.5%)	41 (22.8%)	156 (25.5%)	57 (33.9%)	**.04**
Femoral	321 (33.4%)	38 (21.1%)	216 (35.3%)	67 (39.9%)	**<.01**
Brachiocephalic trunk	197 (20.5%)	46 (25.6%)	128 (20.9%)	23 (13.7%)	**.01**
Carotid	43 (4.5%)	6 (3.3%)	35 (5.7%)	2 (1.2%)	.097

Bold value indicates are statistically significant. *VIT*, Visceral ischemia time; *CPB*, cardiopulmonary bypass; *FET*, frozen elephant trunk; *ET*, elephant trunk.

There was no significant difference among groups in terms of in-hospital death (overall: 114, 12%; group 1 = 15, 8.5%; group 2 = 80, 13.2%; group 3 = 19, 11.3%; *P* = .224), gastrointestinal complications (group 1 = 5%, group 2 = 5.5%, group 3 = 7.1%; *P* = .696), and neurological complications such as stroke (group 1 = 3.3%%, group 2 = 7.2%, group 3 = 4.8%; *P* = .119), transient (group 1 = 15%, group 2 = 16.7%, group 3 = 16%; *P* = .867), and permanent neurological deficits (group 1 = 5.5%, group 2 = 8.9%, group 3 = 6%; *P* = .188) and spinal cord injury (group 1 = 1.7%, group 2 = 2.5%, group 3 = 3.6%; *P* = .537). However, groups 2 and 3 had a higher incidence of respiratory complications (group 1 = 35, 19.4%; group 2 = 172, 28.1%; group 3 = 36, 21.4%; *P* = .027) and transient dialysis (group 1 = 5,2.8%; group 2 = 47,7.8%; group 3 = 19, 11.3%; *P* = .011). Overall renal complications were more frequent in groups 2 and 3, although this did not reach statistical significance (group 1 = 23, 13%; group 2 = 119,19.7%; group 3 = 38, 22.6%; *P* = .056) (Table 3). Exploratory laparotomies with or without intestinal resection were performed in 6 patients (0.6%).

**Table 3:** In-hospital results of the patients according to the visceral ischemia time

Short-term outcomes	Overall (n = 960)	VIT ≤30 min (n = 180)	VIT 30-60 min (n = 612)	VIT >60 min (n = 168)	*P* value
In-hospital death	114 (12.0%)	15 (8.5%)	80 (13.2%)	19 (11.3%)	.224
Bleeding requiring reopening	71 (7.4%)	16 (8.9%)	43 (7%)	12 (7.1%)	.662
Neurological complications	220 (22.9%)	35 (19.4%)	148 (24.2%)	37 (22%)	.399
Permanent neurological deficits	75 (7.8%)	10 (5.5%)	55 (8.9%)	10 (6%)	.188
Stroke	58 (6.1%)	6 (3.3%)	44 (7.2%)	8 (4.8%)	.119
Transient neurological deficits	156 (16.2%)	27 (15%)	102 (16.7%)	27 (16%)	.867
Spinal cord injury	24 (2.5%)	3 (1.7%)	15 (2.5%)	6 (3.6%)	.537
Respiratory complications	243 (25.3%)	35 (19.4%)	172 (28.1%)	36 (21.4%)	**.027**
Renal complications	180 (18.9%)	23 (13.0%)	119 (19.7%)	38 (22.6%)	.056
Permanent dialysis	64 (6.7%)	10 (5.6%)	44 (7.3%)	10 (6.0%)	.679
Transient dialysis	71 (7.4%)	5 (2.8%)	47 (7.8%)	19 (11.3%)	**.011**
Intestinal ischemia	21 (2.2%)	4 (2.2%)	15 (2.4%)	2 (1.2%)	.610
Gastrointestinal complications	55 (5.7%)	9 (5%)	34 (5.5%)	12 (7.1%)	.696

Bold value indicates are statistically significant. *VIT*, Visceral ischemia time.

In our analysis of visceral damage biomarkers 24 and 48 hours postsurgery, as well as peak levels, creatinine (*P* < .01) and GOT (*P* < .01 at 24 and 28 hours, *P* = .024 for the peak level) were significantly higher in groups with longer VIT. Glutamic pyruvic transaminase levels in groups 2 and 3 were significantly higher after 24 and 48 hours (*P* < .01), but this did not occur for peak levels (*P* = .373). Bilirubin levels, conversely, had no significative variation among the 3 groups (*P* = .421, *P* = .143, *P* = .430 at 24, 48 hours, and peak level, respectively) (Table 4).

**Table 4:** Visceral ischemia biomarker levels after surgery according to the visceral ischemia time

Biomarker levels	Overall (n = 960)	VIT ≤30 min (n = 180)	VIT 30-60 min (n = 612)	VIT >60 min (n = 168)	*P* value
Creatinine levels (mg/dL)					
At 24 h	1.3 ± 0.66	1.2 ± 0.72	1.3 ± 0.6	1.5 ± 0.7	**<.001**
At 48 h	1.4 ± 0.9	1.3 ± 0.7	1.4 ± 0.8	1.7 ± 1.1	**<.001**
Peak	2 ± 1.6	1.6 ± 1.4	2.0 ± 1.7	2.2 ± 1.6	**<.001**
GOT levels (U/L)					
At 24 h	120 ± 252	99.1 ± 186.7	127 ± 292.9	117.6 ± 131.1	**<.001**
At 48 h	125.3 ± 292	94.2 ± 228.7	137.6 ± 331.4	115.7 ± 184.7	**<.001**
Peak	231 ± 755.2	162.5 ± 340.3	260 ± 891.8	202.7 ± 508.3	**.024**
GPT levels (U/L)					
At 24 h	57.6 ± 156	42 ± 83.6	60.8 ± 174.1	62.7 ± 146.4	**<.001**
At 48 h	73.9 ± 530	36.4 ± 79.9	91 ± 665.6	53.5 ± 88.2	**<.001**
Peak	149.3 ± 716.5	91.4 ± 192.8	177.6 ± 884.2	111.7 ± 281.1	.373
Bilirubin levels (mg/dL)					
At 24 h	1.7 ± 1.9	1.8 ± 2.9	1.7 ± 1.7	1.7 ± 1.3	.421
At 48 h	1.8 ± 4	2.1 ± 7.7	1.7 ± 2.6	1.5 ± 2	.143
Peak	2.5 ± 3.2	2.3 ± 2.4	2.6 ± 3.5	2.2 ± 2.3	.430

Bold value indicates are statistically significant. *VIT*, Visceral ischemia time; *GOT*, glutamic-oxaloacetic transaminase; *GPT*, glutamic pyruvic transaminase.

**Figure E1:**
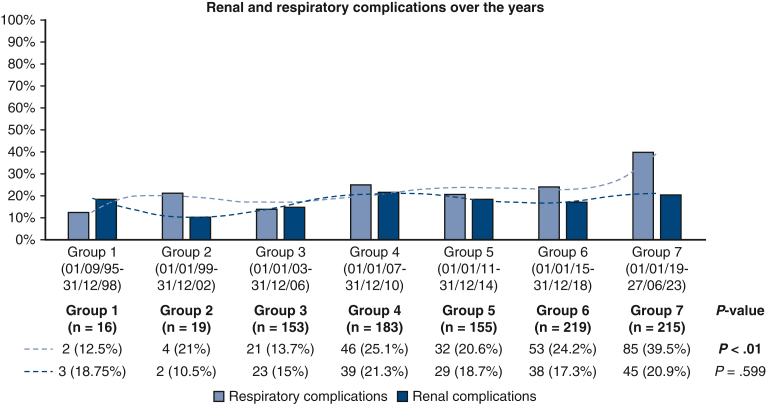
Incidence of renal and respiratory complications in the study population over the study period.

An analysis of the incidence of renal and respiratory complications over the study period can is shown in Figure E1.

### Risk Factors for Renal and Respiratory Complications

The results of the univariate and multivariate analyses are shown in Table 5. The univariate analysis revealed that AKI was significantly linked with preoperative ejection fraction, cardiopulmonary bypass (CPB) time, crossclamp time, renal failure, VIT, and urgency/emergency. These variables preserved their significance in the multivariate analysis, except for crossclamp time, preoperative ejection fraction, and VIT.

**Table 5:** Univariate and multivariate logistic regression analyses for acute kidney injury and respiratory complications

Risk factors	Postoperative AKI		Postoperative respiratory complications	
	Univariate OR (95% CI) *P* value	Multivariate OR (95% CI) *P* value	Univariate OR (95% CI) *P* value	Multivariate OR (95% CI) *P* value
Age	1.01 (0.99-1.03) *P* = .12		**1.02 (1.00-1.03) *P* = .019**	**1.02 (1.01-1.03) *P* = .01**
Sex	0.84 (0.59-1.20) *P* = .33		1.07 (0.79-1.47) *P* = .66	
Ejection fraction	**0.11 (0.014-0.86) *P* = .036**	0.17 (0.019-1.52) *P* = .11	0.2 (0.03-1.29) *P* = .091	
CPB time	**1.01 (1.01-1.01) *P* < .001**	**1.01 (1.01-1.02) *P* = .001**	**1.01 (1.004-1.01) *P* < .001**	**1.01 (1.003-1.01) *P* < .001**
Crossclamp time	**1.01 (1.01-1.01) *P* = .007**	0.99 (0.99-1.00) *P* = .17	**1.01 (1.001-1.01) *P* = .001**	0.99 (0.99-1.003) *P* = .45
Renal failure	**4.42 (2.57-7.58) *P* < .001**	**4.44 (2.52-7.83) *P* < .001**	**3.3 (1.93-5.63) *P* < .001**	**2.98 (1.71-5.21) *P* < .001**
Diabetes	1.18 (0.61-2.29) *P* = .62		1.07 (0.58-1.97) *P* = .82	
Smoking	0.99 (0.71-1.38) *P* = .94		0.98 (0.73-1.32) *P* = .89	
Hypertension	1.31 (0.89-1.92) *P* = .16		1.25 (0.89-1.75) *P* = .18	
Second half of time period	1.11 (0.79-1.57) *P* = .55		**1.67 (1.21-2.31) *P* = .02**	1.35 (0.96-1.91) *P* = .082
Visceral ischemia time	**1.01 (1.01-1.02) *P* = .03**	1.01 (0.99-1.01) *P* = .133	0.99 (0.99-1.01) *P* = .49	
Urgency/emergency	**1.81 (1.30-2.51) *P* < .001**	**3.45 (1.29-2.57) *P* = .001**	**1.84 (1.38-2.48) *P* < .001**	**1.73 (1.27-2.35) *P* < .001**

Bold value indicates are statistically significant. *AKI*, Acute kidney injury; *OR*, odds ratio; *CPB*, cardiopulmonary bypass.

Respiratory complications were significantly related to preoperative age, CPB time, crossclamp time, renal failure, urgency/emergency, and second half of the study period (from 2010). Their significance was confirmed in the multivariate analysis, except for the crossclamp time and second half of the time period.

The fit fractional polynomial plot after cubic spline estimation of the relationship between VIT and postoperative renal complications is depicted in Figure 1. After a rapid decrease, the probability increased slowly along the incrementation of VIT.

Figure 2 shows the probability of respiratory complications over VIT. Maximum level occurred at approximately 50 minutes of VIT, followed by a slow decrease.

## Discussion

Deep hypothermic CA allowed the development of aortic arch surgery, providing brain and visceral organs protection and guaranteeing a bloodless field. However, deep hypothermia soon showed several disadvantages such as prolonged CPB times, which are linked to systemic inflammatory response, coagulopathy and subsequent blood loss,^8^ and temporary neurological dysfunction, particularly regarding higher cognitive functions.^13^^,^^14^

Currently, aortic surgery requiring CA is more commonly performed by associating hypothermia with cerebral perfusion techniques. We observed the same trend toward higher CA temperatures (>25 °C) in our experience, with approximately two-thirds of the study population dating after 2010. The 2022 American College of Cardiology/American Heart Association guidelines recommend cerebral perfusion to improve neurological outcomes (class of recommendation IIa, level of evidence B-NR),^11^ although this recommendation is based on retrospective studies and meta-analysis.

The tendency to perform arch surgery at increasingly higher CA temperatures has been validated in terms of efficacy of cerebral protection if adequate perfusion strategies are applied. The recent GOT-ICE randomized trial showed noninferiority of low-to-moderate hypothermia coupled with cerebral perfusion compared with deep hypothermia in terms of cognitive performance and neuro-imaging techniques.^15^ Much less attention has been placed on the consequences of higher CA temperatures on visceral function.

Previous studies showed visceral protection with mild-to-moderate hypothermia,^16^ highlighting the importance of the duration of the CA, but no specific analysis was performed on the effect of VIT.

The aim of our study was to assess the effect of the duration of CA on visceral function in the context of mild-to moderate hypothermia, including in our analysis cases with VITs even longer than 60 minutes. Most of these patients underwent complex arch procedures with longer operative times, whereas in a few cases long VITs expressed complications and anatomic challenges emerged in the operating room, as in some of the few hemiarch replacements in group 3.

Visceral organ damage can be a complication of any cardiac surgery through gastrointestinal complications^17^ and AKI.^17^, ^18^, ^19^ In the field of aortic surgery and TAAD, however, visceral malperfusion is more common. Both gastrointestinal^20^ and renal complications^21^ are more frequent in arch surgery requiring CA and are linked to worse postoperative outcomes.

Our population proved to be homogenous in terms of preoperative characteristics, except for the obvious fact that groups 2 and 3 included more complex procedures and TAAD.

Surgical treatment was extended to the arch in most patients (67.2% overall), including complex FET or ET procedures (35.5%). Furthermore, our population included many urgent/emergency cases (41.1% overall) and TAAD (34.7% overall).

VITs did not statistically impact short-term outcomes such as in-hospital mortality, stroke and other kinds of neurological complications, intestinal ischemia, and gastrointestinal complications in general. These data prove how careful management of CPB and cerebral protection allow satisfying results.

Respiratory failure is a frequent and feared complication of cardiac surgery linked to worse outcomes in terms of in-hospital mortality and length of stay.^22^ Respiratory complications were statistically more frequent in groups 2 and 3. These types of complications of aortic arch surgery are less investigated in literature, so this result prompted us to perform a cubic spline analysis. Respiratory complications reach a maximum at approximately 50 minutes and then constantly decrease. Although we have no explanation for this at the moment, it might be linked to the fact that group 2 (which comprised patients with VITs ∼50 minutes) included more urgent or emergency cases, which have been linked to higher rates of respiratory tract infections in cardiac surgery.^23^ Urgency/emergency was confirmed as a risk factor for both AKI and respiratory complications in our multivariate analysis.

Renal complications in cardiac surgery, on the contrary, have been widely described and investigated in literature, with a reported incidence between 6% and 21%,^23^, ^24^, ^25^, ^26^ which is in accordance with our data. They have been linked to worse short- and long-term outcomes.^23^^,^^26^ Previous studies showed no difference in the incidence of AKI depending on CA temperatures.^15^^,^^25^^,^^27^^,^^28^

An interesting 2023 study by Hu and colleagues^29^ investigated the link between VIT and postoperative AKI. The authors found an association between VIT more than 40 minutes and AKI in a univariate analysis, but this was not confirmed in the multivariate analysis or once the data were corrected for parameters such as body mass index, preoperative glomerular filtration rate, and nadir CA temperature. Therefore, this study is in accordance with literature and prompted us to further investigate the relationship between renal failure and VIT duration adjusted for CA temperature. In our analysis, overall renal complications had an 18.9% incidence, which increased across groups without reaching significance (*P* = .056). Transient dialysis, on the contrary, was significantly more frequent in groups with higher VITs (*P* = .011). Cubic spline analysis showed how the incidence of renal complications grew constantly with VIT. All of this shows how, with equal CA temperatures, VIT does have an impact on the incidence of renal complications.

Our results confirm the safety of mild-to-moderate hypothermia in terms of visceral complications, although careful cardiopulmonary bypass and cerebral protection strategies are central.

### Study Limitations

Although we were able to include a large population in our study, this comprised patients who underwent surgery over a 28-year period. Changes to surgical strategy and perioperative management might affect outcomes, although we included the influence of the time of surgery in our analysis.

Furthermore, longer VITs were performed in more complex surgeries. Although the baseline characteristics of the groups were homogenous, outcomes might be influenced by more complex aortic pathology leading to longer VIT times. Also, urgent/emergency cases and TAAD were more common in group 2. Some patients might have been affected by some form of subclinical visceral malperfusion without reaching the parameters of malperfusion syndrome.

In the future, further studies on the matter should be performed to better understand the effect of VITs alone on the outcomes of patients undergoing arch surgery under mild-to-moderate hypothermia.

## Conclusions

**Figure 3:**
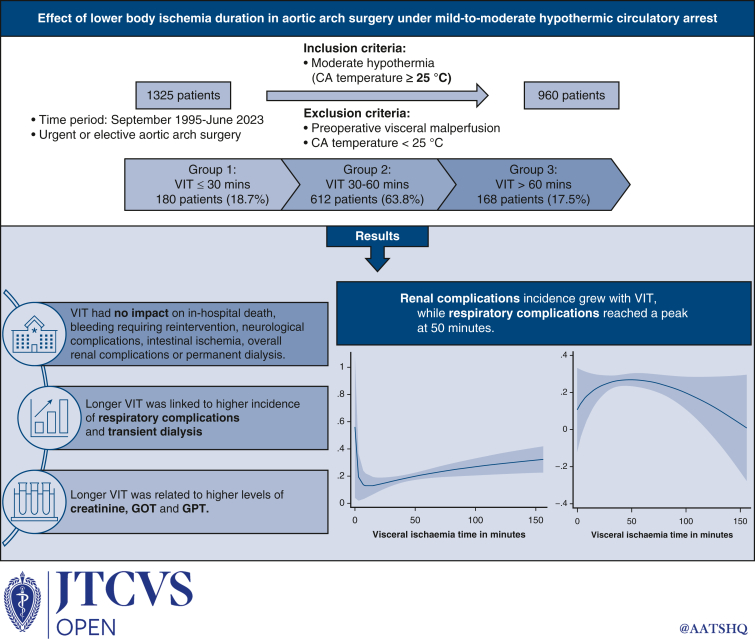
Effect of mild-to-moderate hypothermic circulatory arrest during aortic arch repair.

Mild-to-moderate hypothermia paired with ASCP is a safe visceral organ protection strategy with outcomes comparable to deep hypothermia. However, there is a trend toward a higher incidence of renal complications with longer VITs (Figure 3).

## Conflict of Interest Statement

The authors reported no conflicts of interest.

The *Journal* policy requires editors and reviewers to disclose conflicts of interest and to decline handling or reviewing manuscripts for which they may have a conflict of interest. The editors and reviewers of this article have no conflicts of interest.

## Supplementary Data

Video 1 Video abstract featuring a preview of the study. Video available at: https://www.jtcvs.org/article/S2666-2736(25)00042-7/fulltext.
