# Supplementary figures and images for: Effect of lower-body ischemia duration in aortic arch surgery under mild-to-moderate hypothermic circulatory arrest
Source: JTCVS Open. 2025 Feb 4;24:58–66. doi: 10.1016/j.xjon.2025.01.015 (PMC12039456; doi:10.1016/j.xjon.2025.01.015)

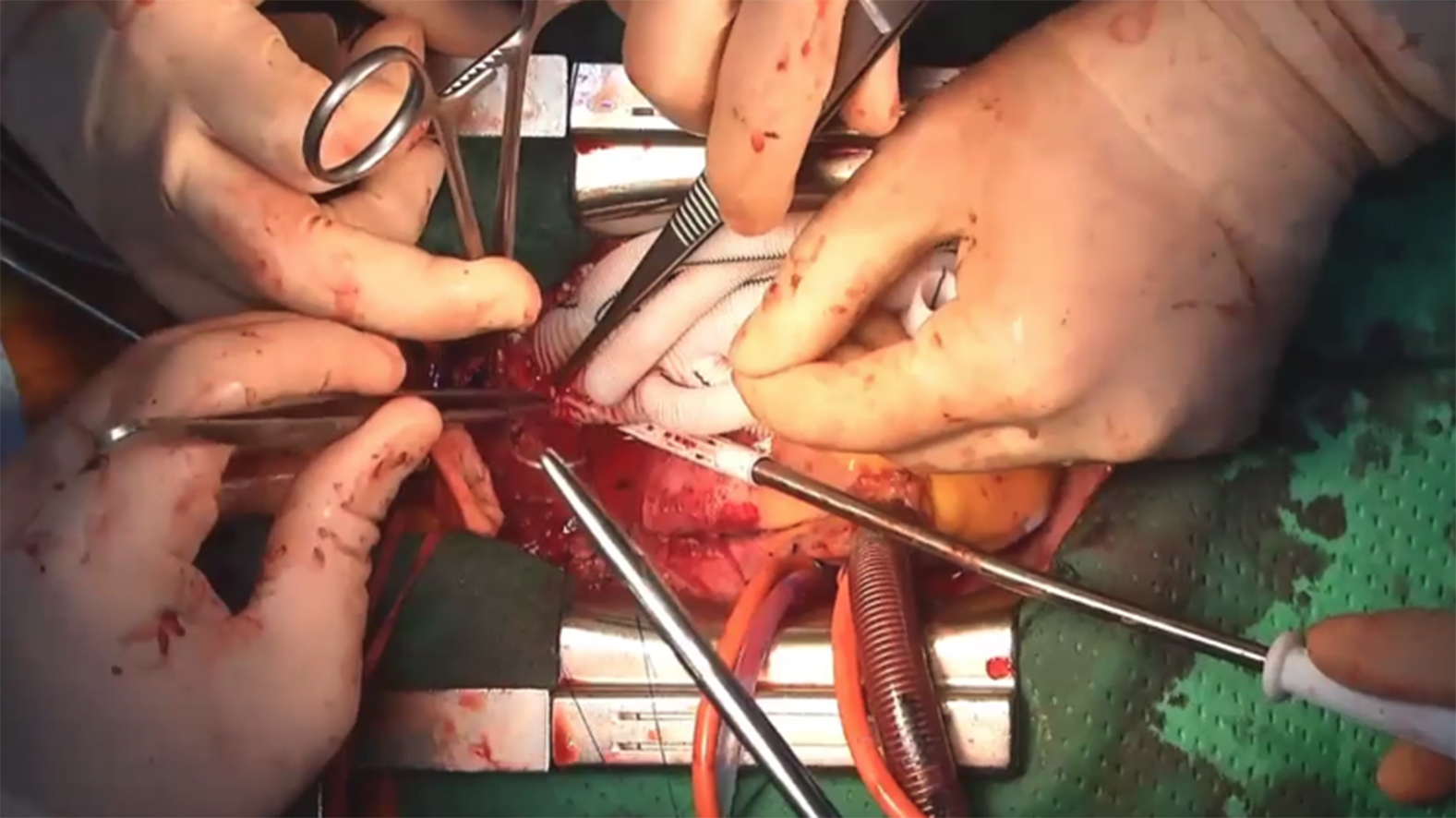

Supplement: Video 1 — Video abstract featuring a preview of the study. Video available at: https://www.jtcvs.org/article/S2666-2736(25)00042-7/fulltext. [file fx2.jpg]
